# Sex Differences in Autoimmune Multimorbidity Across Eleven Disorders: A Real-World Primary Care Study in Germany

**DOI:** 10.3390/medicina61122091

**Published:** 2025-11-24

**Authors:** Karel Kostev, Nimran Kaur, Judith Höfle, Marcel Konrad, Ali Hammed, Christian Tanislav, Ira Rodemer

**Affiliations:** 1 Philipps University, University Hospital, 35037 Marburg, Germany; 2 IQVIA, Epidemiology, 60549 Frankfurt am Main, Germany; 3 IQVIA, Epidemiology, Bangalore 560103, India; 4 IQVIA, Real World Solutions, 60549 Frankfurt am Main, Germany; judith.hoefle@iqvia.com; 5 Health & Social, FOM University of Applied Sciences for Economics and Management, 60486 Frankfurt am Main, Germany; 6 Department of Geriatrics and Neurology, Diakonie Hospital Jung Stilling Siegen, 57074 Siegen, Germany

**Keywords:** autoimmune disorders, multimorbidity, autoimmune multimorbidity, sex differences, Germany

## Abstract

*Background and Objectives*: While most studies have focused on the incidence, pathogenesis, and severity of individual autoimmune diseases (AIDs), limited attention has been given to *autoimmune multimorbidity*—the co-occurrence of multiple AIDs in a single individual. This study aims to examine sex differences in autoimmune multimorbidity across eleven AIDs in a real-world setting. *Materials and Methods*: This retrospective cross-sectional study analyzed data from the Disease Analyzer database (IQVIA) and included 164,596 individuals who visited one of 1037 primary care physicians in 2024. All patients had been diagnosed with at least one of eleven predefined AIDs between 2020 and 2024. For each AID, the prevalence of autoimmune multimorbidity was compared descriptively between female and male patients. *Results*: The total number of patients varied substantially across conditions. The highest numbers were observed for autoimmune thyroiditis (*n* = 51,765), psoriasis (*n* = 39,063), and rheumatoid arthritis (*n* = 33,182), while the lowest numbers were observed for systemic lupus erythematosus (*n* = 897) and Sjögren’s syndrome (*n* = 2728). A notable sex disparity was present in several conditions. For instance, 30.2% of women with systemic lupus erythematosus had at least one additional AID, compared to 25.4% of men; 31.6% of women with Sjögren’s syndrome, compared to 20.7% of men; 28.5% of women with ankylosing spondylitis, compared to 18.6% of men; and 20.7% of women with celiac disease, compared to 12.5% of men. In contrast, autoimmune thyroiditis and rheumatoid arthritis exhibited smaller sex-related differences in autoimmune multimorbidity. Adjusted analyses confirmed these differences after accounting for age and clustering by practice. *Conclusions*: This study reveals significant sex differences in autoimmune multimorbidity among individuals with predefined AIDs in a real-world primary care setting. The findings support the hypothesis that women may be more prone to coexisting autoimmune conditions due to underlying hormonal, genetic, or immunological factors.

## 1. Introduction

Autoimmune diseases (AIDs) are a diverse group of disorders characterized by an abnormal immune response against the body’s own tissues [1]. Collectively, they affect a significant proportion of the global population, with a notable impact on morbidity and healthcare systems. AIDs affect approximately 4.5–10% of people worldwide, and their prevalence has increased in recent decades. There is also significant variation in prevalence by region, sex, and specific disease [2,3]. A striking feature of most AIDs is their considerable sex disparity, with higher rates observed in women (up to 13%) than in men (2.7–7.4%) [3,4].

The biological basis of this sex bias is multifactorial, involving the interplay of hormonal, genetic, immunological, and environmental factors. Sex hormones significantly modulate immune function: estrogens tend to enhance humoral and cell-mediated immunity, whereas androgens and progesterone are more immunosuppressive. Estrogens promote B-cell survival and antibody production, a mechanism that may underlie the female predisposition to antibody-mediated conditions such as systemic lupus erythematosus (SLE) and Sjögren’s syndrome [5,6]. Furthermore, women have two X chromosomes, one of which is typically inactivated; however, incomplete or skewed X-inactivation can result in biallelic expression of immune-related genes, thus exacerbating immune dysregulation [4,6].

While most studies have focused on the incidence, pathogenesis, and severity of individual autoimmune diseases, less attention has been paid to autoimmune multimorbidity, the co-occurrence of multiple autoimmune diseases in the same individual. The phenomenon of autoimmune multimorbidity has significant implications for the diagnosis, treatment, and prognosis of autoimmune diseases [7]. Over the past decade, numerous studies have examined autoimmune multimorbidity in various AIDs [8,9,10,11,12]. While the findings of these studies are clinically relevant, most were conducted outside of Germany and involved small patient samples.

Although several large registry and hospital-based studies have examined the co-occurrence of autoimmune diseases and sex-related effects [3,8], these investigations primarily reflect tertiary care or inpatient populations. Evidence from the primary-care setting, where most autoimmune conditions are first diagnosed and managed, remains limited. The present study addresses this gap by providing a real-world analysis of sex differences in autoimmune multimorbidity using data from over 1000 primary care practices in Germany. In addition, we identify the most frequent co-diagnosed autoimmune diseases for each index condition, separately for women and men, providing new insight into sex-specific multimorbidity patterns.

## 2. Materials and Methods

### 2.1. Data Source

This study utilized electronic medical records from the IQVIA™ Disease Analyzer database, which contains anonymized electronic medical records of outpatients documented in private practices across Germany. The data include baseline demographic variables such as age and sex, as well as physician-recorded diagnoses and prescriptions. The database collects information directly from the computer systems of office-based general practitioners and specialists [13].

The validity and representativeness of the Disease Analyzer database have been evaluated in previous methodological research. Comparisons with national statistics have demonstrated that the database closely reflects the demographic structure, consultation frequencies, and disease prevalence observed in the German outpatient population [13]. Furthermore, the database has been widely used for epidemiological studies in various disease areas, including autoimmune diseases [14,15]. Although specific validation studies for rheumatic diseases are not available, there is no reason to assume that the database is less valid for these conditions. The representativeness of the Disease Analyzer has been demonstrated for demographic characteristics, consultation patterns, and disease prevalence across a wide range of chronic diseases in Germany.

The database currently includes records from approximately 1000 general practices distributed throughout Germany, representing the national outpatient population in terms of age, sex, and regional distribution. Missingness for key variables (age, sex, and diagnosis codes) is below 1%, as these variables are mandatory in the electronic documentation system.

### 2.2. Study Population

This retrospective cross-sectional study included 164,596 adults (aged ≥18 years) who visited one of 1037 primary care physicians in 2024 and had at least one diagnosis of any of eleven predefined autoimmune diseases (AIDs) recorded between 2020 and 2024. The AIDs of interest were rheumatoid arthritis (M05–M06), ankylosing spondylitis (M45), systemic lupus erythematosus (M32), Sjögren’s syndrome (M35.0), psoriasis (L40), type 1 diabetes mellitus (E10), autoimmune thyroiditis (E06.3), Graves’ disease (E05.0), inflammatory bowel disease (K50–K51), celiac disease (K90.0), and multiple sclerosis (G35).

To ensure that analyses reflected currently active patients, only individuals with at least one visit recorded in 2024 were included. Autoimmune diagnoses documented during the preceding five years (2020–2024) were used to identify additional coexisting autoimmune conditions. Thus, the study design reflects a cross-sectional snapshot of autoimmune multimorbidity in 2024, based on a five-year retrospective look-back window, rather than year-by-year prevalence trends.

In the Disease Analyzer database, diagnoses are documented by general practitioners using standardized ICD-10 codes during routine clinical consultations. Diagnoses are typically entered based on the GP’s own evaluation and, when applicable, on diagnostic information provided by relevant specialists such as rheumatologists or dermatologists. Only *confirmed* (“gesichert”) diagnoses were included in this study to ensure diagnostic accuracy. Co-diagnoses were defined as any additional confirmed autoimmune disease recorded during the 2020–2024 look-back period, irrespective of whether the codes were documented on the same or different visit dates. Repeated documentation (≥2 codes) was not required, as this approach could exclude stable chronic conditions seen infrequently in primary care.

**Figure 1:**
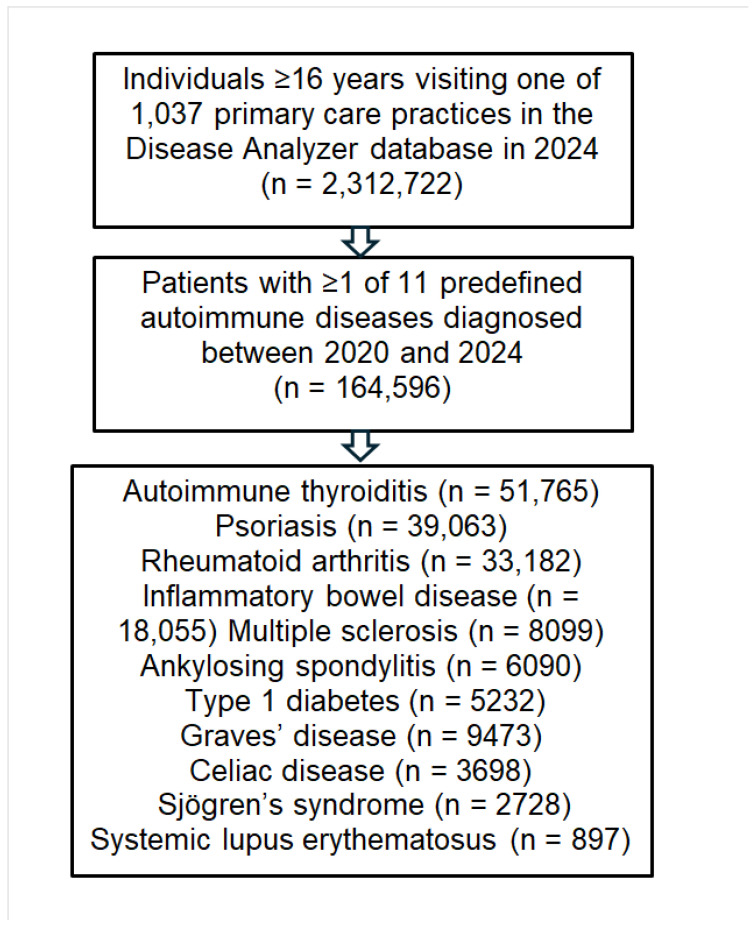
Flow diagram of patient selection and inclusion across eleven autoimmune diseases (AIDs). Study outcome.

A schematic overview of patient selection and disease-specific inclusion is provided in Figure 1.

The primary outcome of this study was the proportion of patients with autoimmune multimorbidity, defined as having at least one additional AID in addition to a predefined AID. Each of the eleven autoimmune diseases was analyzed separately as the primary condition. For each disease, all patients diagnosed with that condition were identified, and the presence of at least one additional autoimmune disease was considered autoimmune multimorbidity. Thus, patients with multiple autoimmune diseases could appear in more than one subgroup. For example, a patient with both systemic lupus erythematosus and psoriasis would be included in both the SLE and psoriasis groups, with the alternate condition counted as the additional AID.

The proportions of additional AIDs were compared descriptively between female and male patients for each AID. Additionally, for each AID, the most frequently co-diagnosed AID was identified and reported separately for each sex.

### 2.3. Statistical Analyses

Chi-squared tests were conducted to compare the proportions of autoimmune multimorbidity between women and men, and a two-sided *p*-value of <0.05 was considered statistically significant. All other analyses were descriptive and did not involve hypothesis testing. The median number of autoimmune diseases per patient and interquartile range (IQR) were calculated.

Multivariable logistic regression analyses were conducted to examine the association between sex and autoimmune multimorbidity, defined as the presence of at least one additional autoimmune disease (≥1 other AID). Models were adjusted for age, modeled as a non-linear term using restricted cubic splines, and accounted for clustering by practice using robust standard errors.

As all patients were observable in 2024, calendar year was not included as a covariate. Separate models were estimated for each of the eleven index autoimmune diseases. Odds ratios (ORs) and 95% confidence intervals (CIs) were calculated for female versus male patients. To account for multiple testing across the eleven index diseases, *p*-values were adjusted using the Benjamini–Hochberg false discovery rate (FDR) procedure.

All analyses were performed using SAS version 9.4 (SAS Institute, Cary, NC, USA).

## 3. Results

### 3.1. Baseline Characteristics

Table 1 summarizes the demographic characteristics of the study population by AID. The number of patients varied substantially across conditions, with the highest numbers observed for autoimmune thyroiditis (*n* = 51,765), followed by psoriasis (*n* = 39,063) and RA (*n* = 33,182). The lowest patient numbers were recorded for SLE (*n* = 897) and Sjögren’s syndrome (*n* = 2728).

Sex distribution revealed notable differences. Diseases with a strong female predominance included autoimmune thyroiditis (85.1%), SLE (86.0%), Graves’ disease (79.7%), and Sjögren’s syndrome (77.2%). In contrast, a slightly higher proportion of male patients was observed in T1D (59.1% male) and AS (55.6% male). The mean age also varied by condition, with the youngest cohort being celiac disease patients (mean age 46.5 years, SD 18.2), and the oldest being RA patients (mean age 65.3 years, SD 15.4).

### 3.2. Autoimmune Multimorbidity by Disease and Sex

**Figure 2:**
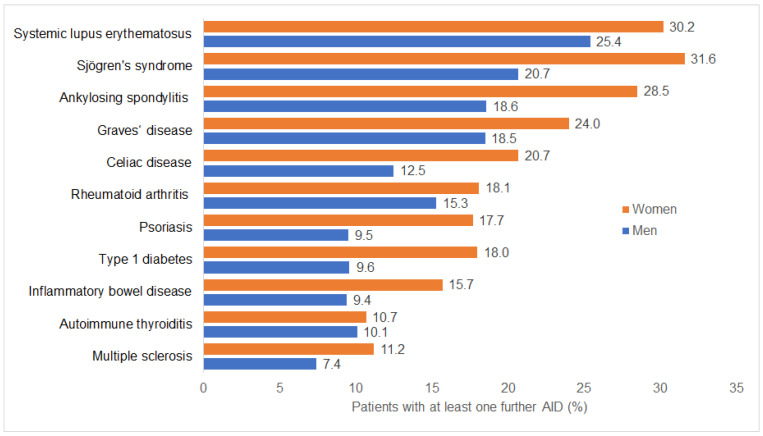
Proportion of patients with at least one additional autoimmune condition by primary condition and sex.

Figure 2 shows the proportion of patients with at least one additional AID, stratified by sex. Across the total study population, the median number of autoimmune diseases per patient was 0 (interquartile range 0–1), reflecting that most individuals had only a single autoimmune diagnosis recorded during the study period. Women consistently exhibited higher rates of autoimmune multimorbidity than men. Notable differences were observed in several conditions. For example, among patients with SLE, 30.2% of women had at least one additional AID, compared to 25.4% of men. In AS, 28.5% of women and 18.6% of men had a co-diagnosis. In Sjögren’s syndrome, another autoimmune condition affected 31.6% of women and 20.7% of men. Additionally, 18.0% of women and 9.6% of men with T1D had at least one other AID.

All depicted sex differences were statistically significant at *p* < 0.05 in unadjusted χ^2^ tests.

### 3.3. Most Frequent Coexisting Autoimmune Disorders

Table 2 shows the most common co-diagnosed autoimmune diseases for each primary condition, stratified by sex. Across all AID conditions, the most common coexisting autoimmune disorders were RA and autoimmune thyroiditis. Among psoriasis patients, 7.9% of women and 4.7% of men had coexisting RA. Conversely, among RA patients, psoriasis was more frequently co-diagnosed in men (8.4%) than in women (7.1%). For SLE patients, RA was the most common frequent coexisting condition, affecting 17.5% of women and 10.3% of men. Among individuals with celiac disease, autoimmune thyroiditis was a coexisting comorbidity, observed in 11.0% of women and 2.9% of men. Similarly, in T1D, coexisting autoimmune thyroiditis was more prevalent in women (7.7%) than in men (2.2%). Graves’ disease and Sjögren’s syndrome also showed high rates of coexisting autoimmune thyroiditis and RA, respectively, with notably higher rates observed in women (15.7% vs. 10.3%, and 14.0% vs. 8.8%).

A comprehensive co-occurrence matrix of all autoimmune disease pairs, showing the absolute number of patients with overlapping diagnoses, is provided in Supplementary Table S1.

In multivariable logistic regression models adjusting for age and clustering by practice, female sex was associated with higher odds of autoimmune multimorbidity for most index diseases (Table 3). After correction for multiple testing using the false discovery rate (FDR) procedure, these associations remained statistically significant for psoriasis, rheumatoid arthritis, inflammatory bowel disease, multiple sclerosis, celiac disease, ankylosing spondylitis, type 1 diabetes, Graves’ disease, and Sjögren’s syndrome. Adjusted odds ratios (female vs. male) ranged from 1.29 to 2.15 across diseases. Full estimates and FDR-adjusted *p*-values are presented in Table 3.

In a sensitivity analysis excluding patients with concurrent psoriasis and rheumatoid arthritis codes—potentially reflecting miscoding of psoriatic arthritis—the overall pattern of sex differences in autoimmune multimorbidity remained unchanged. The adjusted odds ratios for female versus male sex were nearly identical to those in the main analysis, indicating that potential psoriatic arthritis miscoding did not materially influence the findings.

## 4. Discussion

This large, population-based study of over 160,000 adult patients with AIDs examined the prevalence and patterns of autoimmune multimorbidity. The findings highlight a substantial and consistent sex disparity, with women bearing a disproportionate burden of autoimmune multimorbidity. Our findings extend prior large-scale registry analyses by providing an outpatient perspective on autoimmune multimorbidity. Unlike hospital or claims databases, the Disease Analyzer captures diagnoses made and documented in primary care, offering complementary insight into disease clustering as it presents in routine clinical practice.

The predominance of AIDs in women is well established and aligns with previous research. Women account for approximately 78–80% of all autoimmune cases, outpacing men by a ratio of about 2:1 overall [4,16].

Our study builds on this knowledge by showing that women not only have higher rates of individual AIDs but also experience notably higher rates of autoimmune multimorbidity across all disease categories. For instance, among patients with T1D, women exhibit approximately double the rates of additional autoimmune diagnoses compared to men (18.0% vs. 9.6%). This finding is consistent with data from a U.S. study, in which 27.0% of the 179,248 individuals with T1D had at least one additional AID, with higher rates observed in women (35.6%) than in men (19.2%) [17]. More broadly, female patients account for the majority of cases of multimorbidity in AIDs, with estimates placing 85% or more of multiple autoimmune cases in women [18].

The patterns of co-occurrence described in this study are consistent with recognized epidemiological associations. For example, autoimmune thyroiditis frequently occurred alongside celiac disease, and RA was the most common comorbidity in both psoriasis and SLE. The overlap between RA and psoriasis may reflect the presence of psoriatic arthritis (PsA), which is a distinct inflammatory arthritis that shares clinical features with both conditions [19]. A meta-analysis of 266 studies involving almost one million patients revealed that approximately 25% of individuals with psoriasis develop PsA [20].

Icen et al. analyzed a cohort of 603 incident RA patients over 25 years. They found that 15.5% developed ≥4 SLE features, meeting classification criteria for lupus overlap syndrome [21]. However, to the best of our knowledge, no further epidemiological studies investigating the comorbidity of SLE and RA have been published in the last two decades.

Interestingly, around 15% of women and 9% of men with AS had a co-diagnosis of RA in the present study, making RA the most common autoimmune comorbidity among AS patients. A cross-sectional analysis of U.S. insurance claims by Simon et al. found that, of 286,601 RA patients, 3.2% had also been diagnosed with AS [22]. However, no large-scale studies have yet specifically reported on the prevalence of RA among AS patients, highlighting a gap in the literature.

In the present study, around 16% of women and 10% of men with Graves’ disease had a co-diagnosis of autoimmune thyroiditis. The simultaneous presentation of Graves’ disease and Hashimoto’s disease is considered rare and is mostly reported through case studies [23].

However, a subset of patients with Graves’ disease may progress to hypothyroidism over time. Notably, a historical study by Tamai et al. showed that around one-fifth of patients transitioned from Graves’ hyperthyroidism to hypothyroidism—often due to autoimmune processes—over a follow-up period of 0.5 to 10 years [24].

It is important to emphasize that, although several studies have described the comorbidity of different AIDs, few have provided evidence on sex differences in comorbidity. This makes the current study particularly important. The etiological basis for female predominance in autoimmunity is multifactorial. Key mechanisms include hormonal regulation and genetic factors [25]. Furthermore, life transitions including puberty, pregnancy, and menopause can significantly impact immune function, thereby increasing female-specific risk [18]. These biological insights may explain why our data show a higher incidence not only of individual diseases, but also of multiple autoimmune conditions clustering in women.

In addition to descriptive analyses, we conducted multivariable logistic regression to account for age differences and clustering by practice. These models confirmed that female sex was associated with a higher likelihood of autoimmune multimorbidity across most autoimmune diseases, independent of age. Adjustment for age using restricted cubic splines had only a modest impact on effect estimates, suggesting that sex differences were not solely attributable to age structure. After correction for multiple testing using the false discovery rate (FDR), the associations for psoriasis, rheumatoid arthritis, inflammatory bowel disease, multiple sclerosis, celiac disease, ankylosing spondylitis, type 1 diabetes, Graves’ disease, and Sjögren’s syndrome remained statistically significant. This consistency across analytic approaches supports the robustness of the observed sex patterns in autoimmune multimorbidity.

The co-occurrence of rheumatoid arthritis codes among patients with psoriasis or ankylosing spondylitis may partly reflect historical or diagnostic overlap with psoriatic arthritis. Because psoriatic arthritis shares clinical features with both psoriasis and rheumatoid arthritis, miscoding in routine practice is possible. To address this, we conducted a sensitivity analysis excluding patients with concurrent psoriasis and rheumatoid arthritis codes, which did not alter the overall results. Nevertheless, some degree of differential misclassification cannot be ruled out, particularly as women are more frequently labeled with rheumatoid arthritis diagnoses in general practice, which could contribute to a slight overestimation of female multimorbidity.

### 4.1. Clinical Implications

The high prevalence of autoimmune multimorbidity highlights the need for greater clinical awareness of this situation, particularly among female patients. In patients diagnosed with one AID, particularly women, clinicians should maintain a low threshold for screening for coexisting AIDs. Early identification of coexisting AIDs can facilitate timely intervention and improve long-term outcomes. Furthermore, recognizing sex-specific risk patterns can support the development of individualized monitoring strategies and proactive risk mitigation tailored to female patients. Future longitudinal studies should evaluate progression patterns, temporal clustering, and the potential mechanistic pathways underlying sex-based multimorbidity in AIDs. As the present study used a cross-sectional design, it captures coexistence of autoimmune diseases over the observation period but not the timing of their initial diagnoses. Therefore, our results reflect ‘ever diagnosed’ multimorbidity rather than co-clustering at disease onset. Longitudinal analysis would be needed to explore whether simultaneous versus sequential onset reflects shared genetic predisposition or secondary factors such as treatment effects.

### 4.2. Strengths and Limitations

This study has several notable strengths. First, it is based on a large, representative dataset comprising over 160,000 adult patients from more than 1000 general practices in Germany, ensuring high external validity and generalizability of findings to the outpatient population. Second, using real-world electronic medical records from the IQVIA™ Disease Analyzer database allowed for a comprehensive and pragmatic evaluation of autoimmune multimorbidity, minimizing the selection bias inherent in tertiary care or registry-based studies. Third, by focusing on sex-based differences in autoimmune multimorbidity, the study addresses a significant gap in the literature, adding a novel layer of epidemiological insight with important clinical implications for screening and risk stratification.

Despite these strengths, several limitations must be acknowledged. First, the cross-sectional design precludes any causal inference regarding the temporal sequence or development of autoimmune multimorbidity. Because the analysis was based on a prevalent cohort, left truncation and survivor bias cannot be excluded. Patients with long-standing autoimmune diseases have had more time to accumulate additional autoimmune diagnoses, which may lead to higher apparent multimorbidity rates compared with more recently diagnosed patients. Second, diagnoses were identified using ICD-10 codes recorded by general practitioners, which may be subject to misclassification or underdiagnosis, particularly in less symptomatic or early-stage autoimmune diseases. In the Disease Analyzer database, all diagnoses are entered by physicians using standardized ICD-10 codes and only *confirmed* (“gesichert”) diagnoses were included to improve diagnostic accuracy. Although the database has been validated for demographic and epidemiologic representativeness, specific validation metrics for each autoimmune ICD-10 code are not available. Third, the database primarily captures primary-care encounters, whereas several autoimmune diseases—particularly those with severe or complex manifestations—are predominantly managed in specialist settings. As a result, patients treated exclusively in tertiary or hospital care may be underrepresented, while milder or stable cases seen by general practitioners may be relatively overrepresented. Fourth, the database lacks information on disease severity, lifestyle factors, and family history, all of which are important confounders in AID risk and progression. Fifth, while the data are representative of German outpatient care, the results may not be fully generalizable to hospitalized populations or healthcare systems in other countries with different diagnostic practices or disease prevalence. Finally, the definition of autoimmune multimorbidity was limited to eleven predefined autoimmune diseases chosen for their coding reliability and prevalence in primary care. Other autoimmune disorders (e.g., vitiligo, autoimmune hepatitis, autoimmune gastritis) were not included, which may have led to an underestimation of the overall burden of multimorbidity and introduced modest variation in disease-mix across index cohorts. Nevertheless, these limitations are unlikely to explain the consistent and robust sex-specific patterns observed.

## 5. Conclusions

This study reveals significant sex differences in autoimmune multimorbidity among individuals with predefined AIDs in a real-world primary care setting. The findings suggest that women may be more susceptible to overlapping autoimmune conditions, due to hormonal, genetic, or immunological factors. These results highlight the importance of routinely monitoring for multiple autoimmune diagnoses, especially among female patients, to enable earlier detection and more personalized care strategies.

## Figures and Tables

**Table 1:** Baseline characteristics of study patients.

Disease	Number of Patients	Age (Mean, SD)	Female (N, %)	Male (N, %)
Psoriasis	39,063	59.2 (16.9)	20,194 (51.7)	18,869 (48.3)
Rheumatoid arthritis	33,182	65.3 (15.4)	22,649 (68.3)	10,533 (31.7)
Systemic lupus erythematosus	897	54.1 (16.6)	771 (86.0)	126 (14.0)
Autoimmune thyroiditis	51,765	54.1 (16.5)	44,039 (85.1)	7726 (14.9)
Inflammatory bowel disease	18,055	53.2 (18.0)	9757 (54.0)	8298 (46.0)
Multiple sclerosis	8099	52.7 (15.7)	5653 (69.8)	2446 (30.2)
Celiac disease	3698	46.5 (18.2)	2689 (72.7)	1009 (27.3)
Ankylosing spondylitis	6090	57.2 (16.1)	2706 (44.4)	3384 (55.6)
Type 1 diabetes	5232	58.9 (19.3)	2142 (40.9)	3090 (59.1)
Graves’ disease	9473	57.7 (16.8)	7550 (79.7)	1923 (20.3)
Sjögren’s syndrome	2728	64.8 (16.2)	2106 (77.2)	622 (22.8)

**Table 2:** Most frequent coexisting autoimmune disorder among patients with predefined autoimmune disorders.

Disease Affected by Autoimmune Multimorbidity	Most Frequent Coexisting AID	Proportion of Patients with a Co-Diagnosis (%)	
		Women	Men
Psoriasis	Rheumatoid arthritis	7.9	4.7
Rheumatoid arthritis	Psoriasis	7.1	8.4
Systemic lupus erythematosus	Rheumatoid arthritis	17.5	10.3
Autoimmune thyroiditis	Rheumatoid arthritis	3.1	1.9
Inflammatory bowel disease	Rheumatoid arthritis	5.0	2.8
Multiple sclerosis	Autoimmune thyroiditis	4.5	0.9
Celiac disease	Autoimmune thyroiditis	11.0	2.9
Ankylosing spondylitis	Rheumatoid arthritis	14.5	8.7
Type 1 diabetes	Autoimmune thyroiditis	7.7	2.2
Graves’ disease	Autoimmune thyroiditis	15.7	10.3
Sjögren’s syndrome	Rheumatoid arthritis	14.0	8.8

**Table 3:** Multivariable logistic regression of autoimmune multimorbidity (≥1 other autoimmune disease) by index autoimmune disease.

Disease	OR (Females vs. Males (95% CI))	*p* Value, FDR
Psoriasis	2.10 (1.97–2.24)	<0.001
Rheumatoid arthritis	1.29 (1.21–1.39)	<0.001
Systemic lupus erythematosus	1.43 (0.93–2.17)	0.1001
Autoimmune thyroiditis	1.09 (1.00–1.19)	0.0515
Inflammatory bowel disease	1.79 (1.62–1.98)	<0.001
Multiple sclerosis	1.56 (1.31–1.86)	<0.001
Celiac disease	1.88 (1.51–2.34)	<0.001
Ankylosing spondylitis	1.74 (1.54–1.95)	<0.001
Type 1 diabetes	2.15 (1.80–2.57)	<0.001
Graves’ disease	1.40 (1.24–1.58)	<0.001
Sjögren’s syndrome	1.76 (1.35–2.29)	<0.001

## Data Availability

The datasets used and analyzed during the current study are available from the corresponding author upon reasonable request.

## Supplementary Materials

The following supporting information can be downloaded at: https://www.mdpi.com/article/10.3390/medicina61122091/s1, Table S1: Co-occurrence matrix of eleven predefined autoimmune diseases (absolute numbers of overlapping diagnoses).
