# Supplementary material for: Sex Differences in Autoimmune Multimorbidity Across Eleven Disorders: A Real-World Primary Care Study in Germany
Source: Medicina (Kaunas). 2025 Nov 24;61(12):2091. doi: 10.3390/medicina61122091 (PMC12734685; doi:10.3390/medicina61122091)
Supplement: Supplementary file 1 [file medicina-61-02091-s001.zip › medicina-3835501-supplementary.pdf]

Table S1. Co-occurrence matrix of eleven predefined autoimmune diseases (absolute numbers of overlapping diagnoses).

Primary AID ↓ / Coexisting AID →	Psoriasis	RA	SLE	Autoimmune thyroiditis	IBD	MS	Celiac disease	AS	T1D	Graves' disease	Sjögren-Syndrom
Psoriasis		2488	40	1481	688	193	121	401	168	301	182
RA	2488		148	1520	725	162	113	684	159	335	350
SLE	40	148		74	25	7	3	6	4	9	45
Autoimmune thyroiditis	1481	1520	74		527	278	326	194	233	1382	222
IBD	688	725	25	527		108	103	254	60	122	42
MS	193	162	7	278	108		25	27	27	68	23
Celiac disease	121	113	3	326	103	25		15	21	46	24
AS	401	684	6	194	254	27	15		23	61	26
T1D	168	159	4	233	60	27	21	23		67	9
Graves' disease	301	335	9	1382	122	68	46	61	67		40
Sjögren-Syndrom	182	350	45	222	42	23	24	26	9	40	

RA-rheumatoid arthritis ; SLE- systemic lupus erythematosus; IBD- inflammatory bowel disease; MS- multiple sclerosis; AS- ankylosing spondylitis; T1D- Type 1 diabetes
